# Exploring the Topography of the Obturator Artery and Corona Mortis: a Detailed Analysis with Surgical Implications

**DOI:** 10.1007/s00192-024-05774-8

**Published:** 2024-04-18

**Authors:** Monika Konarska-Włosińska, Patryk Ostrowski, Alicia Del Carmen Yika, Martyna Dziedzic, Michał Bonczar, Wadim Wojciechowski, Jerzy Walocha, Mateusz Koziej

**Affiliations:** 1 Department of Anatomy, Jagiellonian University Medical College Mikołaja, Kopernika 12, 33-332 Kraków, Poland; 2 Youthoria, Youth Research Organization, Kraków, Poland; 3 Department of Radiology, Jagiellonian University Medical College, Kraków, Poland

**Keywords:** Obturator artery, Pelvis, Internal iliac artery, Surgery, Anatomy

## Abstract

**Introduction and Hypothesis:**

The obturator artery (ObA) is described as a branch of the anterior division of the internal iliac artery. It arises close to the origin of the umbilical artery, where it is crossed by the ureter. The main goal of the present study was to create an anatomical map of the ObA demonstrating the most frequent locations of the vessel’s origin and course.

**Methods:**

In May 2022, an evaluation of the findings from 75 consecutive patients who underwent computed tomography angiography studies of the abdomen and pelvis was performed.

**Results:**

The presented results are based on a total of 138 arteries. Mostly, ObA originated from the anterior trunk of the internal iliac artery (79 out of 138; 57.2%). The median ObA diameter at its origin was found to be 3.34 mm (lower quartile [LQ] = 3.00; upper quartile [UQ] = 3.87). The median cross-sectional area of the ObA at its origin was found to be 6.31 mm^2^ (LQ = 5.43; UQ = 7.32).

**Conclusions:**

Our study developed a unique arterial anatomical map of the ObA, showcasing its origin and course. Moreover, we have provided more data for straightforward intraoperative identification of the corona mortis through simple anatomical landmarks, including the pubic symphysis. Interestingly, a statistically significant difference (*p* < 0.05) between the morphometric properties of the aberrant ObAs and the “normal” ObAs originating from the internal iliac artery was found. It is hoped that our study may aid in reducing the risk of serious hemorrhagic complications during various surgical procedures in the pelvic region.

## Introduction

The obturator artery (ObA) is described as a branch of the anterior division of the internal iliac artery. It arises close to the origin of the umbilical artery, where it is crossed by the ureter. It courses antero-inferiorly on the lateral wall of the pelvis and passes between the obturator nerve and vein. During its trajectory, the ObA gives off numerous branches, typically classified into two groups: those within the pelvis (pelvic branches) and those extending into the thigh (extrapelvic branches). The pelvic branches of the ObA consist of the following: the iliac branch, which provides vascular supply to the iliac bone and iliacus muscle, forming anastomoses with the iliolumbar artery; the vesical artery, which is responsible for supplying the medial region of the urinary bladder; and the pubic branch, which originates near the obturator canal, traversing the pubic bone, and anastomosing with its contralateral counterpart and with the pubic branch of the inferior epigastric artery. Moreover, the extrapelvic branches encompass:

1. The anterior branch, which courses along the inner edge of the inferior pubic ramus, forming anastomoses with the posterior branch of the femoral artery and the medial circumflex femoral artery. It mainly supplies blood to the obturator externus muscle, hip adductors, and the skin covering the inner thigh.
2. The posterior branch, which is responsible for supplying the muscles that are attached to the ischial tuberosity, such as the ischiocavernosus muscle.
3. The acetabular branch, which courses through the ligament of the head of the femur to supply the femoral head. Typically, this branch originates from the posterior branch [1, 2].

The anatomy of the ObA exhibits a high degree of variability, especially regarding its origin. The ObA can arise from most parts and branches of the internal iliac system, the inferior epigastric artery, or the external iliac artery (referred to as an aberrant ObA), and, although rare, the femoral artery [2, 3]. Furthermore, the anastomosis between the external iliac/inferior epigastric arteries and ObA, known as the corona mortis, garners significant clinical interest. This is primarily because of the potentially life-threatening hemorrhage that can occur from accidental injury to this structure during various pubic surgical procedures [4, 5]. Nevertheless, having adequate knowledge regarding the general topography of the ObA is also highly significant in numerous pelvic and reconstructive surgeries [6, 7].

Although the anatomy of the said artery has been analyzed extensively by various systematic reviews and meta-analyses [7, 8], no study has to our knowledge comprehensively showcased the topography of the ObA and corona mortis in the pelvic region. Therefore, the main goal of the present study was to create an arterial anatomical map of the ObA demonstrating the most frequent locations of the vessel’s origin and course. Moreover, morphometric features of the ObA and the corona mortis will be presented. We hope that our results may help surgeons to create a mental map of the artery during various pelvic surgical procedures, reducing possible iatrogenic injury to these anatomical structures.

## Materials and Methods

### Approvement of the Bioethical Committee

The research protocol was submitted for evaluation and approved by the Jagiellonian University Bioethical Committee, Cracow, Poland (1072.6120.254.2022). The research was conducted in accordance with the allowed criteria throughout the subsequent phases.

### Study Group

In May 2022, an evaluation of the findings from 75 consecutive patients who underwent computed tomography angiography (CTA) studies of the abdomen and pelvis at the Radiology Department of Jagiellonian University Medical College in Cracow, Poland, was performed. Each CTA was evaluated bilaterally; therefore, a total of 150 sides were initially evaluated. The exclusion criteria were defined as trauma to the abdominal or pelvic region that could impact the structure or dimensions of the ObA or its nearby anatomy; substantial artifacts hindering the accurate imaging and measurement of the ObA or its adjacent anatomical region; poor-quality and unreadable images; and significant lack of filling of the whole arterial system with contrast material. If any of the mentioned defects only impacted half of the CTA without affecting the opposite side, the assessment of the other ObA was conducted independently. Most (*n* = 10) of the excluded sides were not analyzed owing to significant artifacts. Another two were disqualified to ensure bias prevention, given that their images were of poor quality. Finally, a total of 138 sides met the inclusion criteria.

### Results Acquisition

All included CTAs were performed on a 128-slice scanner (Philips Ingenuity CT, Philips Healthcare). The main CT imaging parameters were collimation/increase: 0.625/0.3 mm; tube current: 120 mAs; field of view: 210 mm; matrix size: 512 × 512.

All patients received intravenous administration of contrast material at a dose of 1 ml/kg (standard dose). A non-ionic contrast medium (CM) containing 350 mg of iodine per milliliter was used (Jowersol 741 mg/ml, Optiray®, Guerbet, France). The acquisition of CT data was initiated using a real-time bolus tracking technique (Philips Healthcare), with the region of interest (ROI) placed in the descending aorta. CM was injected intravenously using a power injector at a flow rate of 5 ml/s. This was immediately followed by injecting 40 ml of saline solution at the same flow rate. Following injection of CM and saline, image acquisition was automatically started with a 2-s delay when the attenuation trigger value reached a threshold of 120 Hounsfield units (HU). Scanning was performed in the caudocranial direction.

The CTAs were analyzed on a dedicated workstation in the Anatomical Department of Jagiellonian University Medical College, Cracow, Poland. To ensure the highest possible quality of the visualizations and measurements and to minimize potential bias, Materialise Mimics Medical version 21.0 software (Materialise NV, Leuven, Belgium) was used. Three-dimensional (3D) reconstructions of each scan were developed, employing a set of settings, adjusted to each scan.

### Evaluation and Measurements

At the beginning of every examination, each ObA was completely visualized. Following that, a series of measurements in each ObA were taken by two separate authors, and an average was established by considering both sets of results. All measurements were rounded to two decimal places. Cohen’s kappa coefficient analysis of the results of the two raters was performed. Only the results with a score of 0.90 and higher were included in the final analysis. Morphometric features of the ObA and its associated anatomical area were gathered in 30 categories:

1. ObA diameter at its origin (with division into ObA originating from the anterior trunk of the internal iliac artery/posterior trunk of the internal iliac artery and aberrant ObA)
2. ObA cross-sectional area at its origin (with division into ObA originating from the anterior trunk of the internal iliac artery/posterior trunk of the internal iliac artery and aberrant ObA)
3. ObA angle at its origin
4. Diameter of the anterior trunk of the internal iliac artery/posterior trunk of the internal iliac artery near the origin of the ObA
5. Cross-sectional area of the anterior trunk of the internal iliac artery/posterior trunk of the internal iliac artery near the origin of the ObA
6. Distance from the origin of the anterior trunk of the internal iliac artery/posterior trunk of the internal iliac artery to the origin of the ObA
7. Distance from the ObA to the middle anorectal artery
8. Distance from the ObA to the superior vesical artery
9. Distance from the ObA to the uterine artery
10. Distance from the ObA to the inferior vesical artery/vaginal artery
11. Distance from the ObA to the inferior gluteal artery
12. Distance from the ObA to the internal pudendal artery
13. Distance from the origin of the ObA to the origin of the pubic branch
14. Diameter of the pubic branch at its origin
15. Cross-sectional area of the pubic branch at its origin
16. Distance from the origin of the ObA to the origin of the first iliac branch
17. Diameter of the first iliac branch at its origin
18. Cross-sectional area of the first iliac branch at its origin
19. Distance from the origin of the ObA to the origin of the first vesical branch
20. Diameter of the first vesical branch at its origin
21. Cross-sectional area of the first vesical branch at its origin
22. Distance from the origin of the ObA to the origin of the acetabular branch
23. Diameter of the acetabular branch at its origin
24. Cross-sectional area of the acetabular branch at its origin
25. Distance from the origin of the ObA to the origin of the anterior branch
26. Diameter of the anterior branch at its origin
27. Cross-sectional area of the anterior branch at its origin
28. Distance from the origin of the ObA to the origin of the posterior branch
29. Diameter of the posterior branch at its origin
30. Cross-sectional area of the posterior branch at its origin

Nevertheless, after initial statistical evaluation, the distances from the superior vesical artery and the inferior vesical artery/vaginal artery to the uterine artery (8; 10) were excluded from the analyses owing to significant heterogeneity of the results and bias prevention. Furthermore, for the same reason, parameters regarding the first iliac branch, first vesical branch, and acetabular branch (16–24) were also excluded.

Furthermore, in cases in which the corona mortis occurred, an additional set of parameters was gathered: distance from the origin of the corona mortis to the pubic symphysis; and distance from the corona mortis to the superior pubic ramus.

**Fig. 1:**
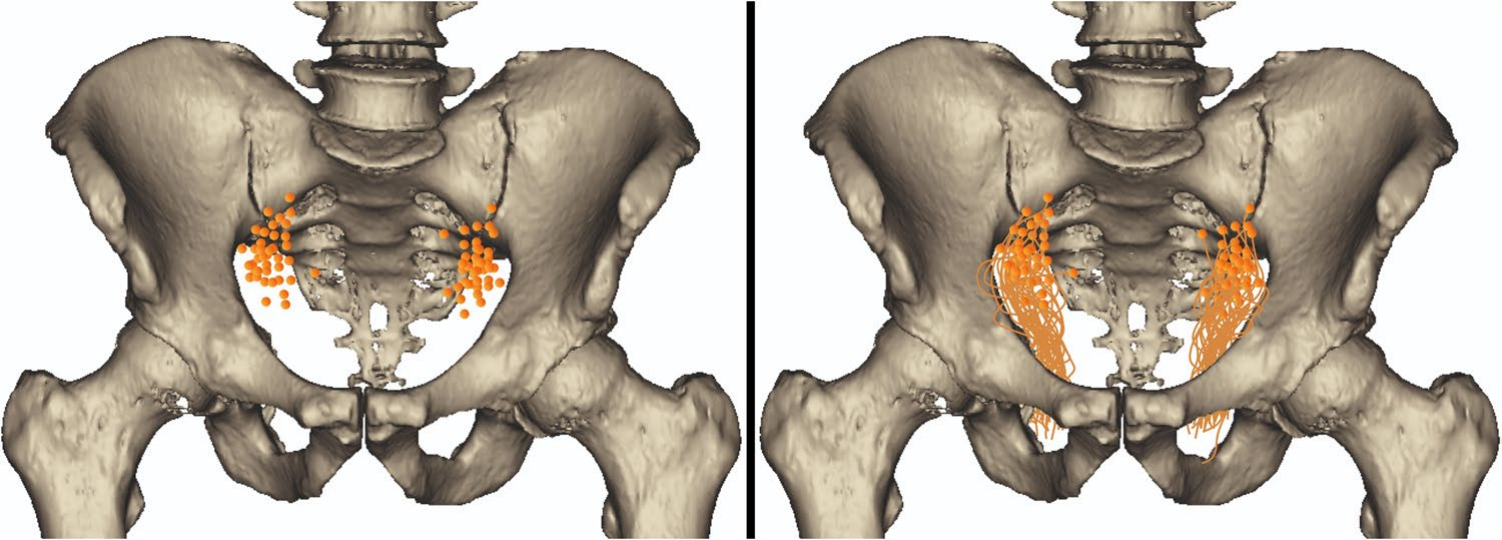
Anatomical map presenting the occurrence of the origin of the obturator artery and its course

**Fig. 2:**
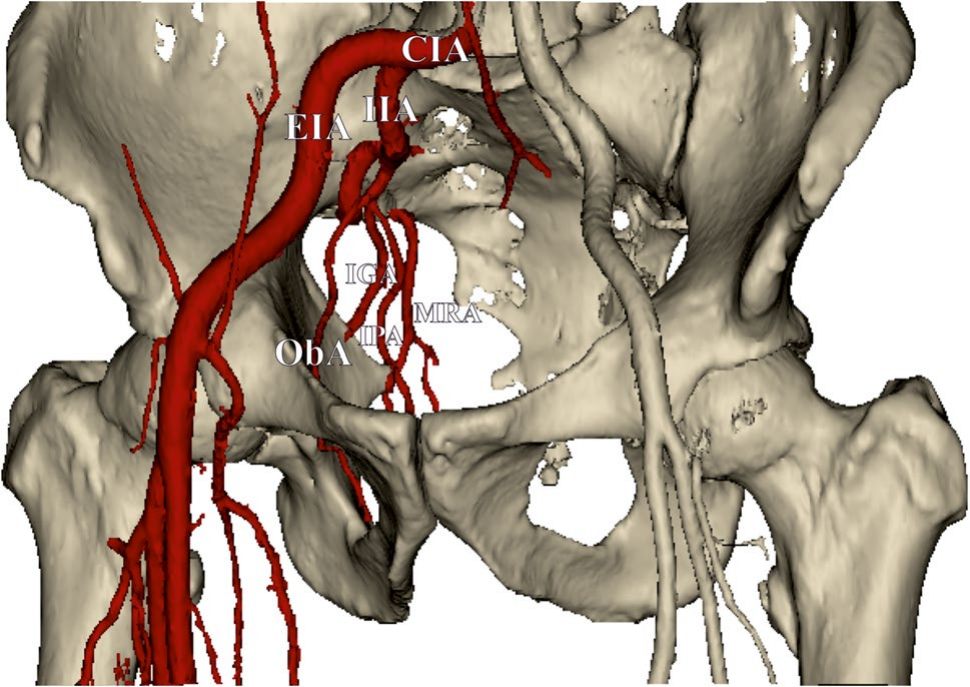
Three-dimensional, computed tomography angiography of the studied area. Some of the arteries were removed in order to provide better visibility. *ObA* obturator artery, *CIA* common iliac artery, *EIA* external iliac artery, *IIA* internal iliac artery, *IGA* inferior gluteal artery, *MRA* middle rectal artery, *IPA* internal pudendal artery

Subsequently, a set of measurements was taken to establish an anatomical map of the occurrence of the origin of the ObA. Using these measurements, a uniform anatomical triangle was defined. Furthermore, the shortest distances from the origins of each ObA to the sides of the triangle were determined. All measurements were taken at a fixed angle to minimize potential bias. Furthermore, the points of origin of each ObA were scaled and applied to the map with respect to the enrolled measurements. A map of the origin of the ObA from the anterior point of view is presented in Fig. 1. The anatomical area of the ObA is presented in Fig. 2.

### Statistical Analysis

Statistical analysis was performed using STATISTICA v13.1 (StatSoft Inc., Tulsa, OK, USA). The frequency and percentages presented qualitative features. The Shapiro–Wilk test was used to assess the normal distribution. Quantitative characteristics were presented by medians and upper and lower quartiles (UQ, LQ), as well as means and standard deviation (SD), depending on the verified normality of the data. Statistical significance was defined as *p* ≤ 0.05. The Mann–Whitney test was used to establish potential differences between groups. The Spearman rank correlation coefficient was used to determine possible correlations between the parameters.

## Results

The presented results are based on a total of 138 sides. The mean age of the patients was 51.8 years (SD = 14.9; minimum = 22.0; maximum = 86.0). All further data refer to the number of sides instead of the number of patients. Seventy-nine (57.2%) of the studied arteries were in women. Mostly, ObA originated from the anterior trunk of the internal iliac artery (79 out of 138; 57.2%). Those and more detailed characteristics of the studied group are gathered in Table 1.

**Table 1:** Qualitative results of the data analysis

Category	*n*	Percentage
Patients’ sex		
Female	79	57.2%
Male	59	42.8%
Patients’ side		
Right	67	48.6%
Left	71	51.4%
Origin of the obturator artery		
ATIIA	79	57.2%
PTIIA	33	23.9%
External iliac system (EIA and IEA)	26	18.8%
- IEA	23	16.7%/88.5%^a^
- EIA	3	2.2%/11.5%^a^

*ATIIA* anterior trunk of the internal iliac artery, *PTIIA* posterior trunk of the internal iliac artery, *IEA* inferior epigastric artery, *EIA* external iliac artery

^a^The first percentage refers to the total number of ObA, whereas the second percentage refers to the number of ObAs originating from the external iliac system

The median ObA diameter at its origin was found to be 3.34 mm (LQ = 3.00; UQ = 3.87). The median cross-sectional area of the ObA at its origin was found to be 6.31 mm^2^ (LQ = 5.43; UQ = 7.32). Furthermore, the median aberrant ObA diameter was 3.01 mm (LQ = 2.73; UQ = 3.29), whereas its median cross-sectional area was 5.10 mm^2^ (LQ = 4.07; UQ = 6.25). The differences in the above-mentioned parameters between “normal” and aberrant ObAs were found to be statistically significant (*p* = 0.00 in both categories).

The median angle of departure of the ObA was found to be 134.11° (LQ = 122.46; UQ = 144.44). Moreover, the median diameter of the anterior trunk of the internal iliac artery at the origin of the ObA was found to be 6.40 (LQ = 5.88; UQ = 7.30). The median distance from the ObA to the middle anorectal artery was set to be 33.77 mm (LQ = 27.63; UQ = 40.24). Furthermore, the median distance from the ObA to the uterine artery was found to be 13.40 mm (LQ = 9.86; UQ = 17.15), whereas the inferior gluteal artery and internal pudendal artery were found to be 44.73 mm (LQ = 38.46; UQ = 50.36) and 16.98 mm (LQ = 9.23; UQ = 25.81) respectively. The median distance from the origin of the ObA to the origin of the pubic branch was found to be 105.19 mm (LQ = 95.34; UQ = 110.88). Detailed results of the analyzed categories are presented in Table 2.

**Table 2:** Results of the measurements

Category	Median	LQ	UQ	Minimum	Maximum	Mean	SD
ObA diameter at its origin (mm)	3.34	3.00	3.87	2.01	4.87	3.44	0.64
ObA cross-sectional area at its origin (mm^2^)	6.31	5.43	7.32	3.68	8.94	6.33	1.18
ObA angle at its origin (°)	134.11	122.46	144.44	100.84	157.34	133.40	14.30
Aberrant ObA diameter at its origin (mm)	3.01	2.73	3.29	1.86	3.98	3.01	0.49
Aberrant ObA cross-sectional area at its origin (mm^2^)	5.10	4.07	6.25	2.15	8.34	5.21	1.61
Diameter of the ATIIA/PTIIA near the origin of the ObA (mm)	6.43	5.89	7.40	4.77	8.94	6.63	0.97
ATIIA	6.40	5.88	7.30	4.77	8.94	6.59	0.99
PTIIA	6.60	6.21	7.46	5.00	8.64	6.73	0.94
Cross-sectional area of the ATIIA/PTIIA near the origin of the ObA (mm^2^)	23.71	19.81	28.02	13.43	38.65	24.20	5.82
ATIIA	24.51	19.58	28.63	13.43	38.65	24.68	6.15
PTIIA	22.97	20.04	25.76	15.54	36.69	23.08	4.85
Distance from the origin of the ATIIA/PTIIA to the origin of the ObA (mm)	17.98	13.64	24.36	3.44	35.45	18.49	6.52
ATIIA	19.23	14.44	24.27	7.40	28.38	18.90	6.04
PTIIA	16.23	11.50	24.74	3.44	35.45	17.55	7.53
Distance from the ObA to the MRA (mm)	33.77	27.63	40.24	18.49	50.42	34.20	7.85
Distance from the ObA to the UA (mm)	13.40	9.86	17.15	3.79	24.18	13.34	4.87
Distance from the ObA to the IGA (mm)	44.73	38.46	50.36	25.17	60.72	44.49	7.81
Distance from the ObA to the IPA (mm)	16.98	9.23	25.81	3.23	84.78	19.97	14.14
Distance from the origin of the ObA to the origin of the pubic branch (mm)	105.19	95.34	110.88	90.15	118.20	104.06	9.87
Diameter of the pubic branch at its origin (mm)	2.19	2.00	2.49	1.89	2.72	2.26	0.27
Cross-sectional area of the pubic branch at its origin (mm^2^)	2.39	2.23	3.77	2.11	4.77	2.85	0.94
Distance from the origin of the ObA to the origin of the anterior branch (mm)	98.48	93.53	105.07	82.46	118.65	99.45	8.97
Diameter of the anterior branch at its origin (mm)	2.46	2.16	2.74	2.00	3.42	2.49	0.35
Cross-sectional area of the anterior branch at its origin (mm^2^)	2.77	2.58	2.96	1.12	3.51	2.74	0.33
Distance from the origin of the ObA to the origin of the posterior branch (mm)	97.67	93.09	104.35	82.46	118.65	99.10	8.97
Diameter of the posterior branch at its origin (mm)	2.19	1.99	2.32	1.70	2.75	2.17	0.22
Cross-sectional area of the posterior branch at its origin (mm^2^)	2.45	2.25	2.67	2.00	3.53	2.49	0.31

*LQ* lower quartile, *UQ* upper quartile, *SD* standard deviation, *ObA* obturator artery, *ATIIA* anterior trunk of the internal iliac artery, *PTIIA* posterior trunk of the internal iliac artery, *MRA* middle rectal artery, *UA* uterine artery, *IGA* inferior gluteal artery, *IPA* internal pudendal artery

Subsequently, potential sexual dimorphism was analyzed. Statistically significant differences between men and women were found to occur in 15 of the studied categories. The median ObA diameter at its origin in women was found to be 3.23 mm (LQ = 2.89; UQ = 3.56), whereas in males, it was set to be 3.68 mm (LQ = 3.15; UQ = 4.30). Median ObA cross-sectional area at its origin in women was found to be 5.67 mm^2^ (LQ = 5.13; UQ = 6.75), whereas in men it was set to be 6.95 mm^2^ (LQ = 6.25; UQ = 7.60). Detailed results with respect to the patient’s sex can be found in Table 3. Furthermore, each parameter was analyzed in order to find potential differences between the patients’ sides. Nevertheless, none of the parameters were found to differ statistically significantly in relation to the studied side.

**Table 3:** Results of the measurements concerning the sex

Category	Sex	Median	LQ	UQ	Minimum	Maximum	Mean	SD	*p* value
ObA diameter at its origin (mm)	Women	3.23	2.89	3.56	2.01	4.43	3.24	0.52	0.00
	Men	3.68	3.15	4.30	2.21	4.87	3.69	0.70	
ObA dross-sectional area at its origin (mm^2^)	Women	5.67	5.13	6.75	4.00	8.94	5.96	1.10	0.00
	Men	6.95	6.25	7.60	3.68	8.84	6.81	1.12	
ObA angle at its origin (°)	Women	136.60	125.49	145.31	100.84	155.20	134.21	14.62	0.45
	Men	133.39	119.83	143.76	103.56	157.34	132.35	13.95	
Aberrant ObA diameter at its origin (mm)	Women	2.98	2.72	3.17	1.86	3.98	2.93	0.51	0.35
	Men	3.13	2.82	3.48	2.39	3.84	3.14	0.46	
Aberrant ObA cross-sectional area at its origin (mm^2^)	Women	4.61	3.84	5.84	2.15	7.91	4.90	1.56	0.22
	Men	5.21	4.61	6.61	3.14	8.34	5.68	1.63	
Diameter of the ATIIA/PTIIA near the origin of the ObA (mm)	Women	6.24	5.69	6.50	4.77	7.69	6.17	0.73	0.00
	Men	7.27	6.55	7.82	4.90	8.94	7.23	0.93	
ATIIA	Women	6.00	5.62	6.39	4.77	7.60	6.01	0.61	0.00
	Men	7.30	6.65	7.88	4.90	8.94	7.25	0.94	
PTIIA	Women	6.36	5.77	7.29	5.00	7.69	6.48	0.84	0.04
	Men	7.23	6.44	7.74	5.29	8.64	7.17	0.96	
Cross-sectional area of the ATIIA/PTIIA near the origin of the ObA (mm^2^)	Women	20.87	18.00	24.42	14.58	29.46	21.32	4.01	0.00
	Men	28.15	23.94	31.74	13.43	38.65	27.92	5.71	
ATIIA	Women	20.53	18.11	25.47	14.58	28.63	21.31	4.08	0.00
	Men	28.77	24.62	32.90	13.43	38.65	28.52	5.89	
PTIIA	Women	21.10	17.42	23.59	15.54	29.46	21.34	3.96	0.01
	Men	25.69	22.36	29.33	20.04	36.69	26.13	4.90	
Distance from the origin of the ATIIA/PTIIA to the origin of the ObA (mm)	Women	16.89	13.64	21.02	3.44	35.45	17.31	6.20	0.02
	Men	21.10	13.55	26.03	5.26	28.38	20.03	6.65	
ATIIA	Women	17.32	14.44	22.11	7.40	28.37	17.84	5.73	0.07
	Men	20.29	14.30	25.84	9.34	28.38	20.10	6.23	
PTIIA	Women	14.88	13.22	18.01	3.44	35.45	16.26	7.06	0.20
	Men	23.59	11.44	26.46	5.26	28.10	19.82	8.10	
Distance from the ObA to the MRA (mm)	Women	35.50	29.99	41.19	18.49	50.42	35.26	8.05	0.11
	Men	31.04	26.86	39.41	20.42	49.30	32.92	7.49	
Distance from the ObA to the IGA (mm)	Women	44.62	38.62	50.65	25.17	60.72	44.94	8.15	0.62
	Men	45.00	37.41	50.01	30.23	56.37	43.91	7.38	
Distance from the ObA to the IPA (mm)	Women	18.10	13.15	31.38	5.87	84.78	23.84	15.76	0.00
	Men	13.30	7.08	19.14	3.23	40.42	14.96	9.81	
Distance from the origin of the ObA to the origin of the pubic branch (mm)	Women	108.52	100.69	116.09	95.34	118.20	107.89	8.89	0.17
	Men	97.40	90.36	106.29	90.15	108.34	98.32	9.35	
Diameter of the pubic branch at its origin (mm)	Women	2.45	2.20	2.55	2.00	2.72	2.40	0.26	0.07
	Men	2.06	1.95	2.15	1.89	2.18	2.05	0.13	
Cross-sectional area of the pubic branch at its origin (mm^2^)	Women	3.12	2.44	3.90	2.23	4.77	3.27	1.03	0.04
	Men	2.22	2.12	2.33	2.11	2.34	2.22	0.12	
Distance from the origin of the ObA to the origin of the anterior branch (mm)	Women	104.08	98.83	111.06	82.46	118.65	103.87	9.65	0.00
	Men	95.20	90.47	99.36	83.28	107.04	95.52	6.14	
Diameter of the anterior branch at its origin (mm)	Women	2.16	2.12	2.31	2.00	2.67	2.22	0.16	0.00
	Men	2.70	2.51	2.93	2.05	3.42	2.74	0.29	
Cross-sectional area of the anterior branch at its origin (mm^2^)	Women	2.64	2.44	2.78	2.22	3.12	2.63	0.24	0.00
	Men	2.88	2.73	3.01	1.12	3.51	2.84	0.37	
Distance from the origin of the ObA to the origin of the posterior branch (mm)	Women	104.08	98.83	112.43	82.46	118.65	104.08	9.85	0.00
	Men	94.70	90.47	98.09	83.28	106.88	95.11	5.72	
Diameter of the posterior branch at its origin (mm)	Women	2.20	2.01	2.33	1.70	2.75	2.17	0.23	0.83
	Men	2.18	1.98	2.32	1.78	2.51	2.16	0.21	
Cross-sectional area of the posterior branch at its origin (mm^2^)	Women	2.45	2.35	2.68	2.00	3.13	2.50	0.28	0.57
	Men	2.42	2.22	2.63	2.00	3.53	2.47	0.33	

*LQ* lower quartile, *HQ* higher quartile, *SD* standard deviation, *ObA* obturator artery, *ATIIA* anterior trunk of the internal iliac artery, *PTIIA* posterior trunk of the internal iliac artery, *MRA* middle rectal artery, *UA* uterine artery, *IGA* inferior gluteal artery, *IPA* internal pudendal artery

*p* values were established using the Mann–Whitney *U* test

*p* values greater than 0.05 were considered statistically significant

Furthermore, potential correlations between patients’ age and studied parameters were established. Statistically significant correlations were found between patients’ age and the diameter of the anterior branch at its origin and the cross-sectional area of the posterior branch at its origin. Detailed results of the correlation analysis can be found in Table 4.

**Table 4:** Correlations between the measured parameters and patients’ age

Category	Spearman’s *R* (patients’ age)
ObA diameter at its origin (mm)	0.00
ObA cross-sectional area at its origin (mm^2^)	0.04
ObA angle at its origin (°)	0.11
Aberrant ObA diameter at its origin (mm)	−0.22
Aberrant ObA cross-sectional area at its origin (mm^2^)	−0.34
Diameter of the ATIIA/PTIIA near the origin of the ObA (mm)	−0.05
ATIIA	−0.06
PTIIA	0.07
Cross-sectional area of the ATIIA/PTIIA near the origin of the ObA (mm^2^)	0.09
ATIIA	0.02
PTIIA	0.29
Distance from the origin of the ATIIA/PTIIA to the origin of the ObA (mm)	−0.09
ATIIA	−0.12
PTIIA	0.04
Distance from the ObA to the MRA (mm)	−0.05
Distance from the ObA to the UA (mm)	−0.07
Distance from the ObA to the IGA (mm)	−0.03
Distance from the ObA to the IPA (mm)	0.17
Distance from the origin of the ObA to the origin of the pubic branch (mm)	−0.37
Diameter of the pubic branch at its origin (mm)	0.49
Cross-sectional area of the pubic branch at its origin (mm^2^)	0.62
Distance from the origin of the ObA to the origin of the anterior branch (mm)	0.10
Diameter of the anterior branch at its origin (mm)	0.25*
Cross-sectional area of the anterior branch at its origin (mm^2^)	0.10
Distance from the origin of the ObA to the origin of the posterior branch (mm)	0.13
Diameter of the posterior branch at its origin (mm)	−0.14
Cross-sectional area of the posterior branch at its origin (mm^2^)	−0.25*

*R* Spearman's correlation test was used in this statistical analysis

*ObA* obturator artery, *ATIIA* anterior trunk of the internal iliac artery, *PTIIA* posterior trunk of the internal iliac artery, *MRA* middle rectal artery, *UA* uterine artery, *IGA* inferior gluteal artery, *IPA* internal pudendal Artery

*p value was less than 0.05

Corona mortis was found to occur in 21.74% of the cases (30 out of 138). The median from the origin of the corona mortis to the pubic symphysis was found to be 62.69 mm (LQ = 58.05; UQ = 67.55). The median from the corona mortis (at the level of the superior pubic ramus) to the pubic symphysis was set to be 48.69 mm (LQ = 44.63; UQ = 56.11). Additionally, a statistically significant difference between men and women has been established in this category (*p* = 0.00). More detailed statistics regarding the corona mortis can be found in Table 5.

**Table 5:** Statistical results of the measurements regarding the corona mortis (30 out of 138; 21.74%

Category	Median	LQ	UQ	Minimum	Maximum	Mean	SD	*p* value
Distance from the origin of the corona mortis to the pubic symphysis (mm)	62.69	58.05	67.55	50.51	70.67	62.21	5.83	–
Women	64.55	59.10	67.50	53.41	70.67	62.92	5.08	0.57
Men	60.83	55.29	68.24	50.51	69.71	61.10	6.96	
Left	62.10	57.84	66.85	50.51	70.67	61.93	5.71	0.56
Right	63.12	58.63	68.34	51.78	69.71	62.58	6.23	
Distance from the corona mortis (at the level of the superior pubic ramus) to the pubic symphysis (mm)	48.69	44.63	56.11	35.76	70.06	50.63	7.84	–
Women	54.54	48.70	58.75	46.03	70.06	55.01	6.62	0.00
Men	44.23	42.53	46.30	35.76	50.02	43.86	3.60	
Left	47.87	44.26	55.51	35.76	62.38	49.36	7.46	0.42
Right	50.61	46.17	57.25	42.67	70.06	52.32	8.34	

*LQ* lower quartile, *UQ* upper quartile, *SD* standard deviation

*p* values were established using Mann–Whitney *U* test

*p* values greater than 0.05 were considered statistically significant

## Discussion

In 1918, Lipshutz stated that "Probably no artery in the human body of proportionate size has so voluminous a literature as the obturator artery" [3]. This statement remains valid even in current times, as various original studies, systematic reviews, and meta-analyses on the anatomy of the ObA have been published in the literature and are continuously being published, showcasing new insights into this subject [5, 7, 8]. The origin of the ObA is remarkably variable, with some studies reporting a rate of variant ObA as high as 26% [8]. Owing to this variability, classification systems aiming to standardize the anatomy of the ObA have been created, with the one presented by Sañudo et al. [9] being the most well known. The said classification system consists of six different types. In type A, the ObA arises from the anterior trunk of the internal iliac artery, whereas in type B, it originates from the inferior epigastric artery. These two variations are those most commonly encountered. Type C involves the ObA originating from the posterior trunk of the internal iliac artery, whereas type D entails the ObA arising from the internal iliac artery prior to its division. The rarest cases fall under type E, where the artery originates from the external iliac artery, and type F, where it arises from the femoral artery. On the basis of this classification, Brachini et al. [7] conducted a systematic review and meta-analysis showcasing the prevalences of ObA variants based on the available data in the literature. The study demonstrated that the most frequent origin of the ObA is from the anterior trunk of the internal iliac artery (61.6%), as described in the major anatomical textbooks. Interestingly, origins such as from the internal pudendal artery (0.9%), the inferior vesical artery (0.1%), and the iliolumbar artery (1%) were also demonstrated, whereas the origin from the femoral artery was never recorded, other than the case reported by Sañudo et al. [9]. Overall, the prevalence of the ObA originating from the internal iliac artery, including its subsequent continuation into anterior and posterior trunks and their branches, was 77.7%, and from the external iliac artery, 22.3%. When the ObA originated from the external iliac artery, also defined as an aberrant ObA [10], the prevalence was divided into two subgroups, namely Sañudo type B (origin from the inferior epigastric artery), being 16.5%, and Sañudo type E (origin from the external iliac artery), being 5.6%. In the present study, our results show that the ObA originated most frequently from the anterior trunk of the internal iliac artery (55.8%), followed by the posterior trunk of the internal iliac artery (23.9%). Interestingly, aberrant ObAs, meaning those originating from the external iliac system, were found in 18.8% of the patients. Among the aberrant ObAs, most originated from the inferior epigastric artery (16.7%) and a small portion directly from the external iliac artery (2.2%). Moreover, our study analyzed the morphometric properties of the analyzed ObAs, and the results of the “normal” ObAs and the aberrant ObAs were compared statistically. A statistically significant difference (*p* < 0.05) was found, and the mean diameter was found to be smaller in the aberrant ObAs (3.01 mm) than in the ObAs originating from the internal iliac artery (3.44 mm).

The vast number of definitions of corona mortis has created discrepancies in the data of studies analyzing this anastomosis [5]. Communicating vessels that course near the superior pubic ramus have been described in different ways, such as aberrant, anomalous, communicating, or variant vessels. Moreover, they are sometimes called pubic branches of the obturator or inferior epigastric vessels [11]. However, it is crucial to note that not all of these vessels establish an anastomosis between the ObA and the external iliac system, and as a result, not all of them can be classified as corona mortis vessels. Therefore, in the present study, we followed the definition used in the meta-analysis conducted by Sanna et al. [5]; namely, that corona mortis is an abnormal anastomosis between the ObA and external iliac or inferior epigastric arteries or veins located behind the superior pubic ramus in the space of Retzius. This excludes aberrant ObA, as they represent ObAs that originate from the external iliac/inferior epigastric arteries but do not form anastomotic channels with the internal iliac system. Corona mortis has also been used to describe venous anastomoses in the retropubic space [5]. The overall prevalence of this anatomical entity, as well as its relationship to other anatomical landmarks, such as the pubic symphysis, have been widely discussed in the past. In the meta-analysis conducted by Sanna et al. [5], corona mortis was found to be present in nearly half of the general population (49.3%), with venous corona mortis (41.7%) being more commonly encountered than arterial corona mortis (17.0%). Moreover, they reported the pooled mean length of the distance from the arterial corona mortis to the pubic symphysis to be 59.90 mm. In our analysis, the corona mortis was found in approximately a quarter of the studied individuals (21.74%). Moreover, the mean distance from the origin of the corona mortis to the pubic symphysis was slightly higher than what was reported by the aforementioned meta-analysis; namely, 62.21 mm. These data can be especially useful in hernia repairs and other surgeries in the pubic region, as laceration of this anastomotic channel may lead to potentially life-threatening hemorrhage.

Owing to the clinical relevance of the ObA and corona mortis, having adequate knowledge about the topography of these anatomical entities is extremely important. Therefore, our study created a novel arterial anatomical map of the ObA, illustrating the location of its origin and course (Fig. 1). Moreover, we provided tools for simple intraoperative localization of the corona mortis using easily palpable landmarks, such as the pubic symphysis (Table 5). Our results may be immensely useful for surgeons performing various gynecological, orthopedic, urological, vascular, and oncological surgeries. During paravaginal repairs, great caution should be taken when dealing with damage to the tendinous arch of the pelvic fascia, as it is closely associated with the corona mortis [5]. Moreover, the presence of vascular variations of the ObA, such as an aberrant ObA and corona mortis, may be a risk factor for significant hemorrhagic complications during fractures of the pubic rami [12, 13]. Importantly, various studies have reported that variations originating from the vascular anatomy of the external iliac, inferior epigastric, or femoral vessels should be acknowledged to avoid severe bleeding during surgical repairs of inguinal and femoral hernias [5, 14]. Given the variable origin, morphometric properties, and topography of the ObA and the potential presence of the corona mortis, it is crucial to perform radiological imaging before the aforementioned surgeries to illustrate and plan the surgical approach adequately.

The present study undoubtedly has several limitations. Some of the parameters that were initially included in the study protocol were excluded owing to potential bias in the results. Nevertheless, those parameters should also be considered and analyzed in further studies, as there is a lack of studies demonstrating the branching pattern of the ObA. Moreover, radiological imaging can only assess arteries that are hemodynamically efficient. Consequently, this is a significant source of bias when evaluating anatomical variations of the ObA and other vascular entities. All participants were white and from the Polish population; therefore, an ethnic analysis was not performed. Furthermore, owing to the lack of data, statistical analysis regarding the patients’ height was not conducted. The authors believe that such analyses should be performed in further studies. Although not without its limitations, the present study attempted to establish detailed morphological and anatomical data on the ObA, meeting the requirements of evidence-based anatomy.

## Conclusion

Our study developed a unique arterial anatomical map of the ObA, showcasing its origin and course. Moreover, we provided more data for straightforward intraoperative identification of the corona mortis through simple anatomical landmarks, including the pubic symphysis. The most common origin of the ObA was from the anterior trunk of the internal iliac artery (57.2%), and the prevalence of the corona mortis was found to be 21.74%. Interestingly, a statistically significant difference (*p* < 0.05) between the morphometric properties of the aberrant ObAs and the “normal” ObAs originating from the internal iliac artery was found. It is hoped that our study may aid in reducing the risk of serious hemorrhagic complications during various surgical procedures in the pelvic region.

## Data Availability

The data that support the findings of this study are available from the corresponding author, upon reasonable request.

## Declarations

### Bioethical Committee

The research protocol was submitted for evaluation and approved by the Jagiellonian University Bioethical Committee, Kraków, Poland (1072.6120.254.2022). The research was conducted in accordance with the permitted criteria throughout the subsequent phases.
